# ﻿Two new species of *Amaurobius* C.L. Koch, 1837 from China (Araneae, Amaurobiidae)

**DOI:** 10.3897/zookeys.1169.102581

**Published:** 2023-07-18

**Authors:** Lu-Yu Wang, Muhammad Irfan, Huan Zhou, Zhi-Sheng Zhang

**Affiliations:** 1 Key Laboratory of Eco-environments in Three Gorges Reservoir Region (Ministry of Education), School of Life Sciences, Southwest University, Chongqing 400715, China Southwest University Chongqing China

**Keywords:** *Amaurobiusguangwushanensis* sp. nov., *Amaurobiuswulongdongensis* sp. nov., description, illustration, morphology, taxonomy

## Abstract

Two new species of the genus *Amaurobius* are described from China: *A.guangwushanensis***sp. nov.** (♂♀) from Sichuan and *A.wulongdongensis***sp. nov.** (♂♀) from Shaanxi. With the addition of two new species, the number of *Amaurobius* species endemic to China now reaches four. Morphological descriptions, photos, and illustrations of copulatory organs, as well as a map of distribution records, are provided.

## ﻿Introduction

*Amaurobius* is the most species-rich genus in the spider family Amaurobiidae Thorell, 1869, comprising 65 species mainly distributed in North America and Europe (WSC 2023). Three European species [*A.erberi* (Keyserling, 1863), *A.fenestralis* (Ström, 1768) and *A.jugorum* L. Koch, 1868] were spread or introduced into west, central, or south Asia. Two Asian endemic species (*A.songi* Zhang, Wang & Zhang, 2018 and *A.spinatus* Zhang, Wang & Zhang, 2018) were described from the northern parts of Chongqing and Sichuan, China (Zhang et al. 2018), suggesting that there should be more local undescribed *Amaurobius* species from China.

Our recent exploration revealed two more new *Amaurobius* species from Sichuan and Shaanxi provinces of China. The four currently known endemic species of *Amaurobius* in China are distributed on the south slope of the Qinling Mountains and the eastern extension of the Hengduan Mountains. Most likely, the uplift of the Qinghai-Tibet Plateau influenced the origin and expansion of this spider group.

## ﻿Materials and methods

All specimens were examined, illustrated, photographed and measured using a Leica M205A stereomicroscope equipped with a drawing tube, a Leica DFC420 camera, and LAS (Leica Application Suite) software (version 4.6). Epigynes were cleared immersing them in pancreatin (Álvarez-Padilla and Hormiga 2007). Leg measurements are rendered here as: total length (femur, patella and tibia, metatarsus, tarsus). All measurements are in millimeters. Morphological terminology follows Zhang et al. (2008). All specimens examined here are deposited in the School of Life Sciences, Southwest University, Chongqing, China (SWUC).

The following abbreviations are used in the text:

Somatic characters: **ALE**–anterior lateral eye; **AME**–anterior median eye; **DTA**–dorsal tibial apophysis; **MOA**–median ocular area; **PLE**–posterior lateral eye; **PME**–posterior median eye; **RTA**–retrolateral tibial apophysis.

Male palp: **Co**–conductor; **DTA**–dorsal tibial apophysis; **eDTA**–exterior branch of DTA; **E**–embolus; **iDTA**–interior branch of DTA; **MA**–median apophysis; **RTA**–retrolateral tibial apophysis; **TA**–tegular apophysis.

Epigyne: **CD**–copulatory ducts; **FD**–fertilization duct; **LT**–lateral teeth; **ML**–median lobe; **S**–spermathecae.

## ﻿Taxonomy

**Family Amaurobiidae Thorell,1869**

**Genus Amaurobius C. L. Koch, 1837** (暗蛛属)

### ﻿ Amaurobius guangwushanensis

Taxon classificationAnimaliaAraneaeAmaurobiidae

sp. nov. (光雾山暗蛛)

60743BB5-C9FA-5101-ACD0-EC0961E28CAE

https://zoobank.org/73040AE8-D0AB-47FE-8390-23A719420954

Figs 1 , 2 , 5

#### Type material.

***Holotype*** ♂: China, **Sichuan Province**: Nanjiang County, Guangwu Mountain, Taoyuan, Sandaoguan, 32°41′15″N, 106°47'45″E, elev. 1377 m, 3.XI.2018, Z.S. Zhang, L.Y. Wang, T. Yuan, L. Yuan & P. Liu leg.; ***Paratypes*** (2♂♂12♀♀): 2♀♀, same data as holotype; 2♂♂3♀♀, Guangwu Mountain, Taoyuan, Lianghekou, 32°40′31″N, 106°46′08″E, elev. 1002 m, 3.XI.2018, Z.S. Zhang, L.Y. Wang, T. Yuan, L. Yuan & P. Liu leg.; 7♀♀, Guangwu Mountain, Taoyuan, 32°41′24″N, 106°47′39″E, elev. 1703 m, 16.V.2013, X.K. Jiang & D. Wang leg.

#### Etymology.

The epithet refers to the type locality (Mt. Guangwu is read as Guangwushan in Chinese); adjective.

#### Diagnosis.

**Figure 1.**
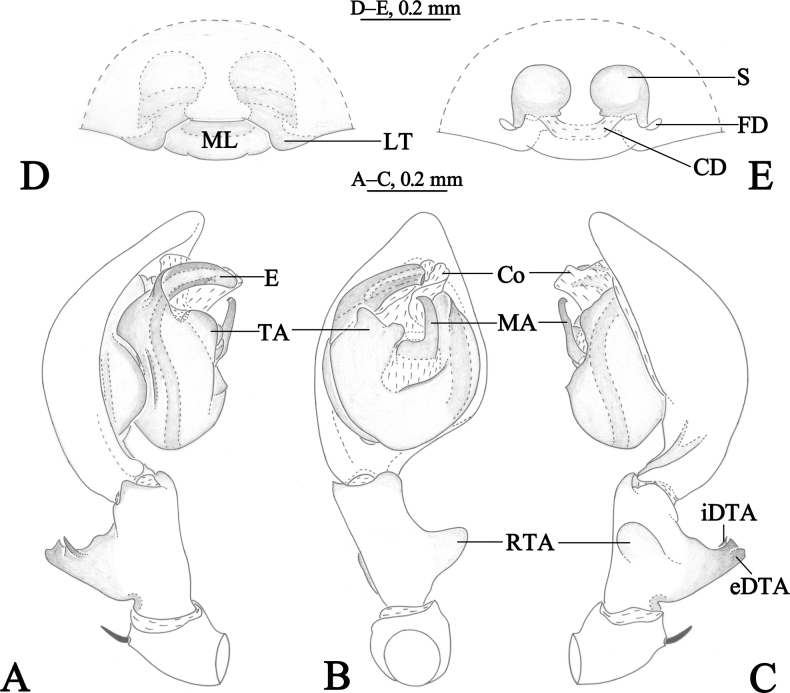
*Amaurobiusguangwushanensis* sp. nov., male holotype (**A–C**) and female paratype (**D–E**). **A** left male palp, prolateral view **B** same, ventral view **C** same, retrolateral view **D** epigyne, ventral view **E** same, dorsal view.

**Figure 2.**
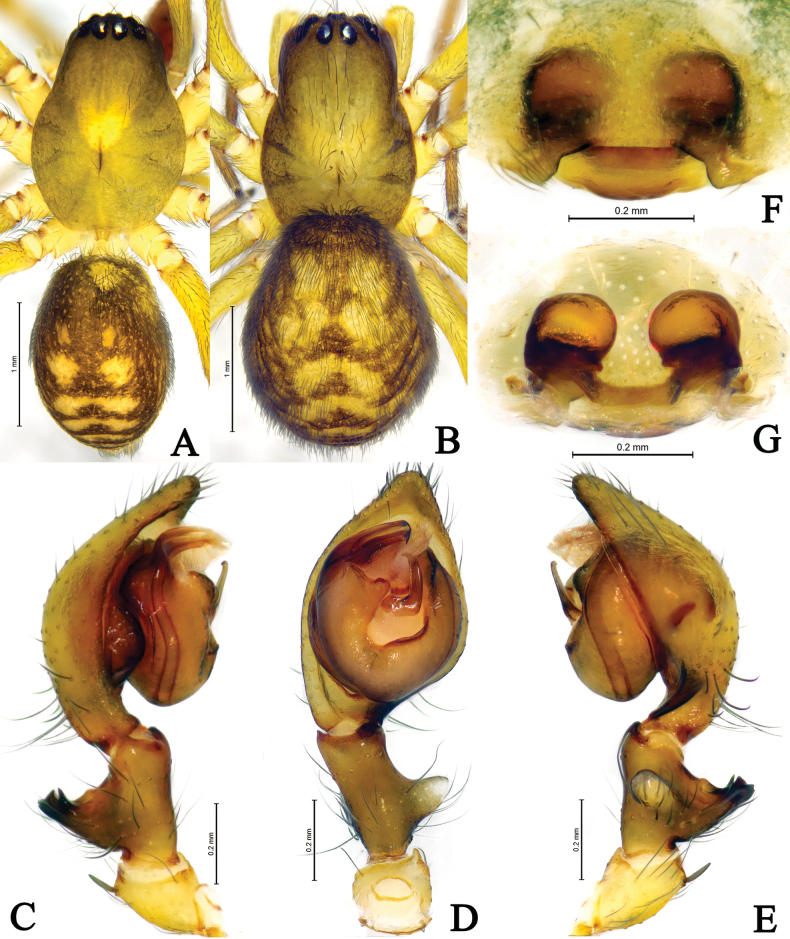
*Amaurobiusguangwushanensis* sp. nov., male holotype (**A, C–E**) and female paratype (**B, F–G**). **A** male habitus, dorsal view **B** female habitus, dorsal view **C** left male palp, prolateral view **D** same, ventral view **E** same, retrolateral view **F** epigyne, ventral view; **G** same, dorsal view.

**Figure 3.**
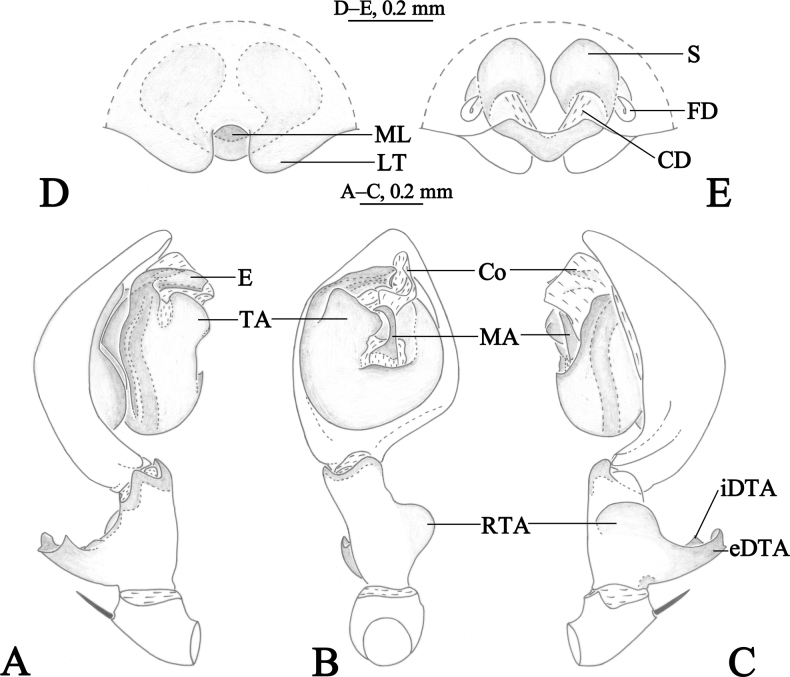
*Amaurobiuswulongdongensis* sp. nov., male holotype (**A–C**) and female paratype (**D–E**). **A** left male palp, prolateral view **B** same, ventral view **C**. same, retrolateral view **D** epigyne, ventral view **E** same, dorsal view.

**Figure 4.**
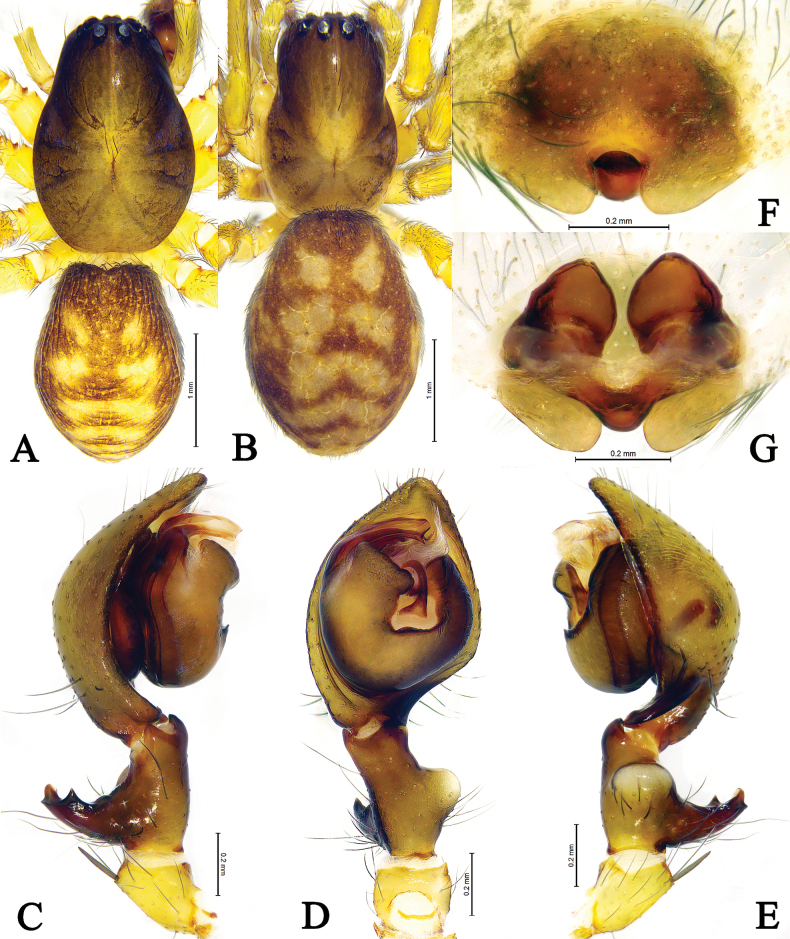
*Amaurobiuswulongdongensis* sp. nov., male holotype (**A, C–E**) and female paratype (**B, F–G**). **A** male habitus, dorsal view **B** female habitus, dorsal view **C** left male palp, prolateral view **D** same, ventral view **E** same, retrolateral view **F** epigyne, ventral view **G** same, dorsal view.

*Amaurobiusguangwushanensis* sp. nov. resembles *A.wulongdongensis* sp. nov. and *A.spinatus* in having a similar embolus in the male [Figs 1A, 2C; see Zhang et al. (2018), figs 4C, 5E, 6A], but can be differentiated by the thumb-shaped retrolateral tibial apophysis in ventral view (Figs 1B, 2D) [vs. somewhat rectangular in *A.spinatus* (see Zhang et al. 2018, figs 4D, 5F); semicircular in *A.wulongdongensis* sp. nov. (Figs 3B, C, 4D, E)]; interior branch of dorsal tibial apophysis somewhat triangular with a sharp pointed end in prolateral view in *A.guangwushanensis* sp. nov. (Figs 1D, 2C) and *A.wulongdongensis* sp. nov. (Figs 3A, 4C) [vs. about thumb-shaped with blunt tip in *A.spinatus* (see Zhang et al. 2018, figs 4C, 5E, 6C); tegular apophysis apex bifurcated in ventral view in *A.guangwushanensis* sp. nov. (Figs 1B, 2D) [vs. broad with small depression at the center in *A.spinatus* (see Zhang et al. 2018, figs 4D, 5F; somewhat rectangular in *A.wulongdongensis* sp. nov. (Figs 3B, 4D)]; median apophysis doorknob-shaped, tip slightly curved in ventral view in *A.guangwushanensis* sp. nov. (Figs 1B, 2D) [vs. hook-shaped in *A.spinatus* (see Zhang et al. 2018, figs 4D, 5F); doorknob-like, tip strongly curved in *A.wulongdongensis* sp. nov. (Figs 3B, 4D)]; conductor apex narrow, apical margin about half the length of the embolus in retrolateral view in *A.guangwushanensis* sp. nov. (Figs 1C, 2E) [vs. broad, apical margin as long as embolus both in *A.spinatus* (see Zhang et al. 2018, figs 4E, 5G) and *A.wulongdongensis* sp. nov. (Figs 3C, 4E)]. The epigyne can be differentiated from the related species by the lateral teeth wider than long with round margin (Figs 1A, 2F) (vs. longer than wide with pointed tip in *A.spinatus* Zhang, Wang & Zhang, 2018, figs 4A, 5C, 6B); median lobe somewhat sheet-like in *A.guangwushanensis* sp. nov. (Figs 1A, 2F) [vs. oval in *A.spinatus* (see Zhang et al. 2018, figs 4A, 5C, 6B); round in *A.wulongdongensis* sp. nov. (Figs 3D, 4F)], lateral teeth c. 1/4 length of median lobe with round tip (Figs 1A, 2F) [vs. about half the length of median lobe, horn-like with tapering tip in *A.spinatus* (see Zhang et al. 2018, figs 4A, 5C, 6B); broad, semicircular, almost two times larger than median lobe in *A.wulongdongensis* sp. nov. (Figs 3D, 4F)].

#### Description.

**Male** (holotype, Fig. 2A). Total length 3.12–3.52. Holotype (Fig. 2A) total length 3.52. Carapace 1.77 long, 1.26 wide; opisthosoma 1.59 long, 1.14 wide. Carapace yellowish. Cervical groove indistinct, radial furrows distinct. Eye measurements and interdistances: AME 0.06, ALE 0.11, PME 0.10, PLE 0.11; AME–AME 0.04, AME–ALE 0.05, PME–PME 0.06, PME–PLE 0.09, ALE–PLE 0.04. MOA 0.31 long, front width 0.14, back width 0.29. Clypeus height 0.11. Chelicerae brown, with 4 promarginal and 4 retromarginal teeth. Endites and labium yellowish brown, longer than wide. Sternum yellowish brown, with brown setae. Legs yellowish. Leg measurements: I 5.88 (1.62, 2.04, 1.35, 0.87); II 4.55 (1.30, 1.50, 1.05, 0.70); III 3.83 (1.11, 1.23, 0.93, 0.56); IV 5.00 (1.42, 1.66, 1.29, 0.63). Opisthosoma oval, dorsum yellowish, with six brown chevrons extending posteriorly, venter yellowish brown.

Palp (Figs 1A–C, 2C–E). Femur almost as long as cymbium. Patella with strong dorso-apical short spine. Tibia with large thumb-shaped retrolateral tibial apophysis, originating near the base of tibia. Dorsal tibial apophysis large, exterior branch of dorsal tibial apophysis longer than wide, c. 1/3 length of tibia, with wavey tip in retrolateral view, interior branch of dorsal tibial apophysis short, somewhat triangular with pointed end in prolateral view. Cymbium longer than both tibia and patellae, with retrolateral angular projection. Bulb oval, slightly longer than wide. Tegulum widest in middle part, tegular apophysis with bifurcated apex. Conductor membranous, sheet-like. Median apophysis sclerotized, doorknob-like, about more than half-length of embolus, present at the center of bulb. Sperm duct visible in prolateral and retrolateral view. Embolus originating prolaterally, short, flat, with a round tip.

**Female** (Fig. 2B). Total length 4.29–4.36. One of paratypes (Fig. 2B) total length 3.84. Carapace 1.52 long, 1.05 wide; opisthosoma 2.40 long, 1.67 wide. Eye measurements and interdistances: AME 0.05, ALE 0.10, PME 0.09, PLE 0.10; AME–AME 0.05, AME–ALE 0.06, PME–PME 0.10, PME–PLE 0.12, ALE–PLE 0.04. MOA 0.30 long, front width 0.15, back width 0.28. Clypeus height 0.12. Legs yellowish brown. Leg measurements: I 4.14 (1.20, 1.46, 0.88, 0.60); II 3.90 (1.05, 1.14, 0.76, 0.50); III 3.09 (0.93, 1.07, 0.68, 0.41); IV 4.06 (1.21, 1.38, 0.97, 0.50). Opisthosoma color pattern as in male.

Epigyne (Figs 1D, E, 2F, G). Median lobe wider than long; lateral teeth with round margin. Copulatory ducts transverse, located between spermathecae. Spermethecae globular, spaced by about half of radius. Fertilization ducts originating posteriorly, slightly curved, directed laterally.

#### Distribution.

**Figure 5.**
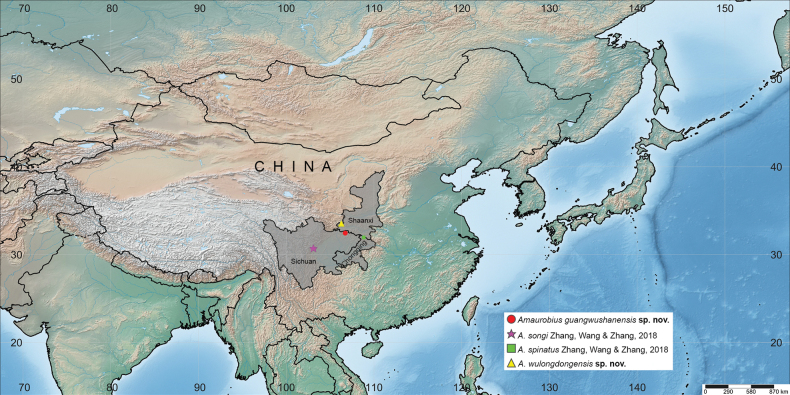
Distribution records of four *Amaurobius* species in China.

Known only from the type locality, Sichuan, China (Fig. 5).

### ﻿ Amaurobius wulongdongensis

Taxon classificationAnimaliaAraneaeAmaurobiidae

sp. nov. (五龙洞暗蛛)

90C3A54D-4BDF-5ACE-96A6-11DBB6C14447

https://zoobank.org/A9F7F1B9-0D48-4895-85A6-32603A937A21

Figs 3 , 4 , 5

#### Type material.

***Holotype*** ♂: China, **Shaanxi Province**: Lueyang County, Wulongdong Forest Park, 33°36′17″N, 106°18′34″E, elev. 1786 m, 17 October 2018, L.Y. Wang leg.; ***Paratypes***: 3♀♀, same data as holotype.

#### Etymology.

The specific name refers to the type locality; adjective.

#### Diagnosis.

See diagnosis of *Amaurobiusguangwushanensis* sp. nov.

#### Description.

**Male** (holotype, Fig. 4A) total length 3.99. Carapace 2.12 long, 1.43 wide; opisthosoma 1.83 long, 1.34 wide. Carapace yellowish brown. Cervical groove and radial furrows distinct. Eye measurements and interdistances: AME 0.05, ALE 0.12, PME 0.11, PLE 0.11; AME–AME 0.05, AME–ALE 0.06, PME–PME 0.12, PME–PLE 0.13, ALE–PLE 0.05. MOA 0.36 long, front width 0.17, back width 0.34. Clypeus height 0.11. Chelicerae dark, with 4 promarginal and 4 or 5 retromarginal teeth. Endites and labium yellowish brown, longer than wide. Sternum yellowish, with brown setae. Legs yellow brown. Leg measurements: I 7.40 (2.01, 2.50,1.87, 1.02); II 5.19 (1.37, 1.85, 1.23, 0.74); III 4.70 (1.40, 1.55, 1.16, 0.59); IV 5.83 (1.74, 2.02, 1.49,0.58). Opisthosoma oval, dorsum yellowish brown, with six brown chevrons, extending posteriorly, venter yellowish brown.

Palp (Figs 3A–C, 4C–E). Femur almost as long as cymbium. Patella with strong dorso-apically short spine. Tibia with large semicircular retrolateral tibial apophysis, originating near the base of tibia. Dorsal tibial apophysis large, exterior branch of dorsal tibial apophysis large, longer than wide, interior branch of dorsal tibial apophysis short, somewhat triangular. Cymbium longer than both tibia and patellae, with retrolateral angular projection. Bulb oval, slightly longer than wide. Tegulum widest in middle part. Conductor membranous, sheet-like. Median apophysis sclerotized, doorknob-like, about more than half-length of embolus, present at the center of bulb. Sperm duct visible in prolateral and retrolateral view. Embolus originating prolaterally, short, flat, with bifurcate tip.

**Female** (Fig. 4B). Total length 4.29–4.36. One of paratypes (Fig. 4B) total length 4.36. Carapace 2.05 long, 1.36 wide; opisthosoma 2.40 long, 1.75 wide. Eye measurements and interdistances: AME 0.07, ALE 0.13, PME 0.12, PLE 0.11; AME–AME 0.07, AME–ALE 0.09, PME–PME 0.12, PME–PLE 0.14, ALE–PLE 0.06. MOA 0.37 long, front width 0.20, back width 0.35. Clypeus height 0.14. Leg measurements: I 5.39 (1.55, 1.84, 1.20, 0.80); II 4.23 (1.24, 1.45, 0.95, 0.59); III 3.60 (1.13, 1.03, 0.91, 0.53); IV 4.79 (1.46, 1.59, 1.19, 0.55). Opisthosoma color pattern as in male.

Epigyne (Figs 3D, E, 4F, G). Median lobe reduced, somewhat round, with lateral teeth large, semicircular. Copulatory ducts V-shaped, located between spermathecae. Spermathecae semiglobular, almost touching each other. Fertilization ducts originating postero-laterally.

#### Distribution.

Known only from the type locality, Shaanxi, China (Fig. 5).

## Supplementary Material

XML Treatment for Amaurobius guangwushanensis

XML Treatment for Amaurobius wulongdongensis

## ﻿Additional information

### Author ORCIDs

Lu-Yu Wang https://orcid.org/0000-0002-5250-3473

Muhammad Irfan https://orcid.org/0000-0003-0445-9612

Huan Zhou https://orcid.org/0009-0004-4507-6241

Zhi-Sheng Zhang https://orcid.org/0000-0002-9304-1789

### Data availability

All of the data that support the findings of this study are available in the main text.
